# Impact of Caffeine on Aquatic Ecosystems: Assessing Trophic-Level Biological Responses

**DOI:** 10.3390/jox15030086

**Published:** 2025-06-03

**Authors:** Sara Rodrigues, Rita S. Alves, Sara C. Antunes

**Affiliations:** 1 Centre Interdisciplinary of Marine and Environmental Research, Laboratory Associated (CIIMAR/CIMAR, LA), University of Porto, Terminal de Cruzeiros do Porto de Leixões, 4450-208 Matosinhos, Portugal; 2 Department of Biology, Faculty of Sciences, University of Porto (FCUP), Rua do Campo Alegre, S/N, 4169-007 Porto, Portugal; rita.sofia.19@hotmail.com

**Keywords:** emerging contaminant, ecotoxicology, freshwater ecosystems, individual and sub-individual responses, species sensitivity distribution

## Abstract

This study evaluates the effects of caffeine (CAF) on the bacteria *Aliivibrio fischeri*, the microalga *Raphidocelis subcapitata*, the macrophyte *Lemna minor*, and the larvae of *Chironomus riparius*, aiming to understand its environmental impact and contribution to ecological risk assessment. Bioluminescence inhibition in *A. fischeri* (EC_50_ = 998.5 mg/L) and growth inhibition in *R. subcapitata* and *L. minor* (EC_50_ = 60.1 mg/L and EC_50_ = 649.2 mg/L, respectively) were observed. For *L. minor*, reduced catalase (CAT) activity and non-linear responses in glutathione S-transferases (GSTs) were recorded. No significant changes were observed in proline, malondialdehyde (MDA), and pigment contents. In *C. riparius*, acute mortality (LC_50_ = 644.5 mg/L) was observed, and growth was significantly affected after 10 days of CAF exposure (EC_50_ = 81.62 mg/L for fresh biomass). After 10 days of exposure, there was an increase in CAT activity and thiobarbituric acid reactive substances, with TBARS levels both at concentrations ≥82.64 mg/L, and a decrease in GSTs (92.18 mg/L) and acetylcholinesterase (AChE) (≤62.09 mg/L) activities of *C. riparius*. The results show that CAF exposure affects organisms’ metabolic and physiological functions, with varying sensitivities among species, potentially leading to ecological disturbances in aquatic ecosystems. The hazardous concentration for 5% of species was 4.42 mg/L. Long-term studies are necessary to understand the risk of caffeine under more realistic scenarios.

## 1. Introduction

Caffeine (CAF) is a chemical compound that naturally occurs in various plants (e.g., coffee plants, *Coffea* spp.) and is found in several products, such as coffee, tea, energy drinks, pharmaceuticals, and hygiene and beauty products [1,2,3]. The caffeine concentrations in these products are very different, ranging from 36 to 804 mg/L in coffee, 17 to 551 mg/L in chocolates, 13 to 68 mg/L in teas, 267 to 340 mg/L in energy drinks, and 1002 to 1353 mg/L in dietary supplements [4,5]. In pharmaceutical and therapeutic medicine, CAF is also widely used as a cardiac, brain, and respiratory stimulant and is often employed as a diuretic [1]. Regarding physical and chemical properties, CAF is relatively stable under environmental conditions (e.g., salinity, light, and temperature), highly resistant to biological and chemical degradation, highly soluble in water (about 20.0 g/L), with a low octanol–water partition coefficient (log K_ow_ = −0.07), and low volatility [6,7]. CAF has a half-life of approximately 100–240 days [2,8], or up to 10 years (under constant temperatures between 8 ᵒC and 20 ᵒC in water, without light, in higher temperature conditions) [9].

CAF spread and increasing concentration in aquatic ecosystems have been observed. Thus, CAF has been considered a pseudo-persistent contaminant [10]. Various studies have already reported high concentrations of CAF (ng/L to µg/L) in different aquatic environments, including freshwater (rivers, lakes) with values ranging between 0.05 µg/L and 33.2 µg/L [11,12], in groundwater with values ranging between 0.01–0.08 µg/L and 0.68 µg/L [1,13], in drinking water (0.50–35 µg/L) [13,14], in reservoirs 27.7 µg/L [15], in brackish and saltwater water (estuaries; seas and oceans) with values between 0.00033 µg/L and 8.23 µg/L [16], and influents and effluents wastewater (0.02 and 86,000 µg/L) [17,18]. The continuous and increasing input of CAF into aquatic environments over the years has also led to its classification as an emerging contaminant and an indicator of anthropogenic pollution in these environments [10,19,20]. Although wastewater treatment plants (WWTPs) achieve high removal rates of CAF from domestic and urban wastewater (Leiria, Portugal—100% [21]; South Korea—92.3% [22]; Southwest United Kingdom—73.9 % [23]; and Slovenia—96.3 % [24]), the growing use of CAF over the years has raised increasing concerns about the contamination of aquatic ecosystems [25].

CAF is not yet listed as a priority substance for monitoring under the Water Framework Directive, according to several responsible organizations, such as the Portuguese Environment Agency (APA), the Organization for Economic Cooperation and Development (OECD), the Environmental Protection Agency (EPA), and the European Chemicals Agency (ECHA). Although CAF is considered pseudo-persistent due to its long half-lives [10], it is generally regarded as a low-priority substance by various entities because of its low bioaccumulation potential and relatively limited harmful effects on aquatic organisms [3,19,26,27,28,29,30,31,32,33,34,35,36]. Caffeine is readily absorbed through biological membranes due to its high-water solubility and low log K_ow_ value [6,7], allowing it to diffuse across cell membranes. In aquatic organisms, it is often absorbed passively through gills or integumental surfaces and distributed systemically [6,7]. Although biotransformation in aquatic invertebrates is less studied, oxidative metabolism via cytochrome P450 enzymes has been reported in fish and molluscs, resulting in metabolites such as paraxanthine, theobromine, and theophylline [2,19,27,28,29,30,31]. In several vertebrates, caffeine is known to inhibit phosphodiesterases, modulate adenosine receptors, and induce oxidative stress, all of which may explain observed biochemical alterations in aquatic species [2,3,15].

CAF is described as a psychoactive drug belonging to the methylxanthines group, which can cause stimulation, cell death, alterations in locomotor activity, changes in cell cycle regulation, and/or oxidative stress in both target and non-target species [16,37]. However, available databases and the literature still provide limited information on the ecotoxicity of CAF for freshwater organisms [26,27]. Nevertheless, over the past decades, ecotoxicological studies on CAF’s effects on non-target organisms have increased, but more research is needed to understand the impacts on different organisms within the aquatic trophic web and those with essential ecological roles in aquatic ecosystems.

Based on this background, we hypothesize that caffeine, despite being considered a low-priority contaminant, can elicit significant biological alterations in non-target aquatic species across multiple trophic levels. The main objective of this study is to investigate the environmental impact of CAF through individual and sub-individual responses in representative freshwater model species, contributing to a more comprehensive ecological risk assessment. For the bacteria *Aliivibrio fischeri* (decomposer), a standard Microtox^®^ test was conducted to evaluate bioluminescence inhibition. Growth inhibition tests were performed with the microalga *Raphidocelis subcapitata* (a primary producer and a key organism in nutrient cycling) and the macrophyte *Lemna minor* (a primary producer that provides habitat). For *L. minor*, biochemical determinations were also carried out: catalase (CAT) and glutathione S-transferases (GSTs) activities, which reflect oxidative stress and detoxification capacity, respectively; proline content as an indicator of oxidative and osmotic stress; and malondialdehyde (MDA) levels, which indicate lipid peroxidation and oxidative damage. Additionally, pigment quantification (total chlorophyll and carotenoids) was also performed. *C. riparius* (an insect larvae that plays a vital role in benthic ecosystems, participating in nutrient cycling and serving as prey for higher trophic levels) were exposed to both acute and simplified emergence assays, the latter involving biochemical determinations: CAT and GSTs activities, levels of thiobarbituric acid reactive substances (TBARS, and indicator of lipid peroxidation), and acetylcholinesterase (AChE) activity. AChE activity serves as a marker for neurotoxicity, as caffeine can modulate neurotransmitter signaling through adenosine receptor antagonism and increased acetylcholine availability [15,30].

## 2. Materials and Methods

### 2.1. Caffeine and Test Concentrations

CAF (CAS: 58-08-2; 1,3,7-trimethylxanthine; chemical formula: C_8_H_10_N_4_O_2_) with a molecular weight of 194.19 g/mol and 99% purity was purchased from Sigma Aldrich (Merck KGaA, Darmstadt, Germany). Stock solutions and tested concentrations were prepared by diluting CAF in the appropriate culture medium for each species (Table 1). The chemical analysis of the CAF concentrations was determined by the lowest and highest CAF concentration tested for each species (Table 1) by EPA Method 538: Determination of Selected Organic Contaminants in Drinking Water by Direct Aqueous Injection–Liquid Chromatography/Tandem Mass Spectrometry (DAI-LC/MS/MS), using a Column Luna Omega Polar C18 100 × 3.0 mm, 3 µm (Phenomenex, Torrance, CA, USA) and Mass Spectrometer Sciex 6500+ Triple Quadrupole (AB Sciex, Framingham, MA, USA). The reference substance to conduct the analysis was CAF (CAS: 58-08-2), and 1 mL was used as an internal standard. An injection of 150 µL of each CAF sample was used for HPLC-MS/MS quantification. The limit of quantification (LOQ) of the equipment was 0.00001 mg/L for CAF.

### 2.2. Organisms, Culture Maintenance, and Bioassays

#### 2.2.1. *Aliivibrio fischeri*

*A. fischeri* is a bioluminescent marine bacterium used as a model organism for acute toxicity assessment through the Standard Microtox^®^ test [38]. The decomposer, being Gram-negative, is a facultative anaerobe bacterium [39], a symbiont with some animals, and widely distributed in nature [40,41]. *A. fischeri* was acquired lyophilized from the company that developed the assay, and the first step to begin the assay is to rehydrate the bacterium in a reconstitution solution [38]. The bioluminescence of *A. fischeri* was measured after 30 min of exposure to a range of CAF concentrations (Table 1), following the protocol outlined in the Microtox^®^ Acute Toxicity Basic Test Procedures manual by Modern Water. The tested concentrations were defined based on previous studies, such as Lomba et al. [34]. The results were expressed as EC_50_ values, along with the corresponding 95% confidence intervals, to report the toxicity values of CAF for *A. fischeri*.

#### 2.2.2. *Raphidocelis subcapitata*

*R. subcapitata* is a microalgae, a primary producer that converts solar energy into organic matter, important for producing oxygen, nutrient cycling, and is sensitive to pollutants in aquatic ecosystems. In this way, impacts on the development of these organisms can have repercussions on higher organisms in the aquatic web [35]. Long-term cultures of *R. subcapitata* were maintained in our laboratory under controlled conditions (24 ± 2 °C and constant light) in Woods Hole MBL medium [42] and renewed once a week, following the methodology by Pinto et al. [43]. Growth inhibition assay for *R. subcapitata* was performed according to the standardized protocol n. 201 [44], with adaptations described in Pinto et al. [43]. The initial concentration of the microalgae was 5 × 10^4^ cells/mL [44]. After 3 days (72 h) of exposure to different concentrations of CAF (Table 1), the absorbance at λ = 440 nm was measured for all replicates using a spectrophotometer (UV-1600PC spectrophotometer, VWR International, Leuven, Belgium). The tested concentrations were defined based on previous studies [33,34,35]. The cell concentration in each well was calculated using the equation: C = (ABS_72h_ − 0.0455)/(4 × 10^−8^), where C is the algae concentration in cells/mL (cell density), and ABS_72h_ is the absorbance measured at the end of the assay (λ = 440 nm). The results were expressed as EC_50_ values, with a 95% confidence interval, and growth rate inhibition was used to calculate the EC_50_, following the formula:

$$\%\;Ir=\frac{µc-µt}{µc}\times 100$$

% *I_r_*: percentage of inhibition of the specific growth rate;

µc: average specific growth rate (μ) in the control group;

µt: average specific growth rate for each replicate of each treatment.

#### 2.2.3. *Lemna minor*

Macrophytes like *L. minor* play a central role in freshwater ecosystems, as several ecological services depend on or are associated with their presence (e.g., primary productivity, molecular oxygen production and release, water flow, habitat, nutrient sources, and phytoremediation) [45]. Long-term cultures of *L. minor* were maintained in our laboratory under controlled conditions (23 ± 1 °C and constant light) in Steinberg medium, and renewed once a week, following the standardized protocol n. 221 [46]. *L. minor* growth inhibition assays were conducted according to OECD protocols, with a few adaptations described by Nunes et al. [45]. *L. minor* was exposed for 7 days to a range of CAF concentrations (Table 1). The tested concentrations were defined based on previous studies, such as Ramírez-Morales et al. [36]. At the end of the assay, the fresh weight of *L. minor* was assessed for each replicate. According to the standardized protocol [46], the results were expressed based on fresh biomass (weight) for a subsequent calculation of the EC_50_ values and 95% confidence intervals. Subsequently, the fronds were washed, dried, and divided into four Eppendorf tubes (4 replicates per treatment), then stored at −80 °C for later evaluation of sub-individual parameters: quantification of photosynthetic pigments (chlorophylls and carotenoids), activities of the enzymes CAT and GSTs, levels of proline, and MDA.

##### Sub-Individual Assessment of *L. minor*

The quantification of photosynthetic pigments was performed in each replicate (~5 mg), where the concentration of total chlorophyll and carotenoids was quantified according to Lichtenthaler [47], with some adaptations indicated in Pinto et al. [43]. Pigment extraction was carried out with 1 mL of 96% ethanol, overnight at 4 °C. The pigment content was expressed per mg of fresh weight.

Additionally, for each replicate, *L. minor* fronds were divided into three portions for biochemical determinations of (1) enzyme activities of CAT and GSTs (~30 mg), (2) MDA levels (~15 mg), and (3) proline content (~10 mg). Due to the reduced growth rate at the highest tested CAF concentration (1000 mg/L), it was impossible to quantify the total chlorophyll, carotenoid concentration, and proline content at this concentration. CAT and GSTs activities, MDA levels, and proline content were determined according to Pinto et al. [43]. Proline content was extrapolated from a calibration curve obtained by measuring the absorbance of standard proline solutions at known concentrations (0.0125–0.2 mg/mL).

#### 2.2.4. *Chironomus riparius*

*C. riparius* are ectothermic organisms [48] commonly used as ecological and ecotoxicological models in various studies [49] due to their wide distribution in freshwater habitats, ease of growth in laboratory conditions, sensitivity to pollutants and contaminants, ability to measure responses at different biological levels and bioindicators of sediment quality and benthic ecosystems [50]. Long-term cultures of *C. riparius* were kept in our laboratory at room temperature (19–21 °C), with a photoperiod of 16 h^light^ and 8 h^dark^, and continuous aeration in larval phases [51]. Total medium (ASTM hard water) and sediment renewal were performed every five days [51]. Every two days, the larvae were fed with ground Tetra Goldfish food (Tetra GmbH, Melle, Germany), while adults were fed with sugar water, following standardized protocol n. 235 from the OECD [51]. After the hatching of the egg masses, the organisms were fed once they reached their first larval stage, according to the specified proportions (0.05 mg of fish food/larvae [51]).

The acute toxicity assessment (24 h) was conducted following standardized protocol nº235 from the OECD [51], during 24 h exposure at a range of CAF concentrations (Table 1). The tested concentrations were defined based on previous studies, such as Moore et al. [3]. After the exposure period, mortality/immobilization was recorded to determine the LC_50_ values. Additionally, a sub-chronic toxicity assessment was performed in a medium sediment using a simplified version (standardized protocol n. 219; [52]) for 10 days. For this, glass jars were used with sediment (2 cm) and culture medium (ASTM hard water) with the defined concentrations of CAF (~8 cm; concentration of CAF; Table 1), maintaining a 1:4 ratio (sediment: medium) [52]. In this experiment, larvae were used that had been separated at the time of hatching and fed until they reached 7 to 10 days of age (larval stage III; [52]). For each concentration and control, 3 replicates were performed, each one with 10 larvae of *C. riparius*. Every 48 h, the organisms were fed, and the jars were sealed with parafilm to avoid medium evaporation. The experiment took place in a climate chamber with controlled temperature conditions (20 ± 1 °C), with a photoperiod of 16 h^light^ and 8 h^dark^, and continuous aeration for 10 days. At the end of the assay, mortality and biomass (fresh weight) of the exposed organisms were evaluated for each replicate. Subsequently, the larvae were divided for biochemical marker quantification: 3 replicates with 3 organisms for AChE activity and 3 replicates with 5/6 organisms for oxidative stress (activities of CAT and GSTs, levels of TBARS). The Eppendorf tubes were stored at −80 °C until the biochemical analyses were performed.

##### Sub-Individual Assessment of *C. riparius*

The biological samples for determining the biochemical markers (CAT and GSTs activities and TBARS levels) were sonicated in 1 mL of phosphate buffer (50 mM, pH 7.0 with 0.1 % Triton X-100) using a sonicator (Microson™ Ultrasonic Cell Disruptor XL; Misonix, Inc., Farmingdale, NY, USA). Then, the homogenates were centrifuged at 14,000 rpm for 10 min at 4 °C. For the quantification of AChE activity, the biological samples were homogenized in 500 µL of phosphate buffer, pH 7.2, at 4 °C, using a sonicator (Microson™ Ultrasonic Cell Disruptor XL; Misonix, Inc., Farmingdale, NY, USA). Then, the samples were centrifuged at 4 °C for 3 min at 6000 rpm. AChE, CAT, and GSTs activities, and TBARS levels were determined according to those described in Diogo et al. [19]. The total protein concentration was determined in all samples according to the methodology described by Bradford [53], adapted for microplates, to express all biochemical biomarkers per mg of protein.

### 2.3. Statistical Analysis

The values of E(L)C_50_ and their respective 95 % confidence intervals (using the delta method) were determined by fitting a nonlinear concentration–response toxicity model (LL3) to the yield data of *R. subcapitata* and *L. minor*, as well as the bioluminescence inhibition data of *A. fischeri*, using the drc package [54] for R software (version 4.2.3). Bioluminescence inhibition and growth were modeled as a continuous variable using a three-parameter logistic model, where the lower asymptotes of the curve were fixed at 0, following Ritz [55]. The estimation of the LC_50_ values for *C. riparius* was performed with the results of dead/immobilized organisms (using the R package “drc”; [54]), using a special case of the log-logistic dose–response model, where the curve asymptotes are fixed at 1 (all organisms are dead/immobilized) and 0 (none are immobilized), following the reasoning of Ritz [55].

After EC_50_ determinations for each species, and according to the classification proposed in EU-Directive 93/677/ECC [56], CAF was classified regarding the toxicity to freshwater species: very toxic (EC_50_ ≤ 1 mg/L), toxic (1 < EC_50_ ≤ 10 mg/L), harmful (10 < EC_50_ ≤ 100 mg/L), and not harmful (EC_50_ > 100 mg/L).

The results of the bioassays and sub-individual parameters obtained were tested for normality using the Shapiro–Wilk test and for the homogeneity of variances using Levene’s test. A one-way ANOVA was conducted on the results obtained, followed by Dunnett’s test whenever significant differences were detected, to discriminate the differences between the tested concentrations of CAF and the control group. All statistical analyses were performed in SPSS Statistics v26, using 0.05 as the significance level.

### 2.4. Species Sensitivity Distribution (SSD)

Species sensitivity distribution (SSD) curves were obtained by combining the acute toxicity data (EC_50_) from this study and the literature. A log-probit distribution was used, modeling the data and allowing the estimation of the confidence intervals at 95% (CI_95%_), through a spreadsheet (US Environmental Protection Agency; https://www.epa.gov/sites/default/files/2017-10/ssd_generator_v1.xlsm; accessed on 19 February 2025). Estimated hazardous concentrations for 5% of species (HC_5_) and respective lower (LL) and upper (UL) values were calculated considering the literature data and the results of the present study in the software ETX 2.3.1 by RIVM (https://rvs.rivm.nl/onderwerpen/risicobeoordeling/modellen-voor-risicobeoordeling/ETX; accessed on 19 February 2025) [57].

## 3. Results

### 3.1. Aliivibrio fischeri

Exposure to CAF induced an increase in the inhibition of bioluminescence in the bacteria *A. fischeri*, with a dose–response effect. Thus, the EC_50_ and respective CI_95%_ are represented in Table 2.

### 3.2. Raphidocelis subcapitata

**Figure 1:**
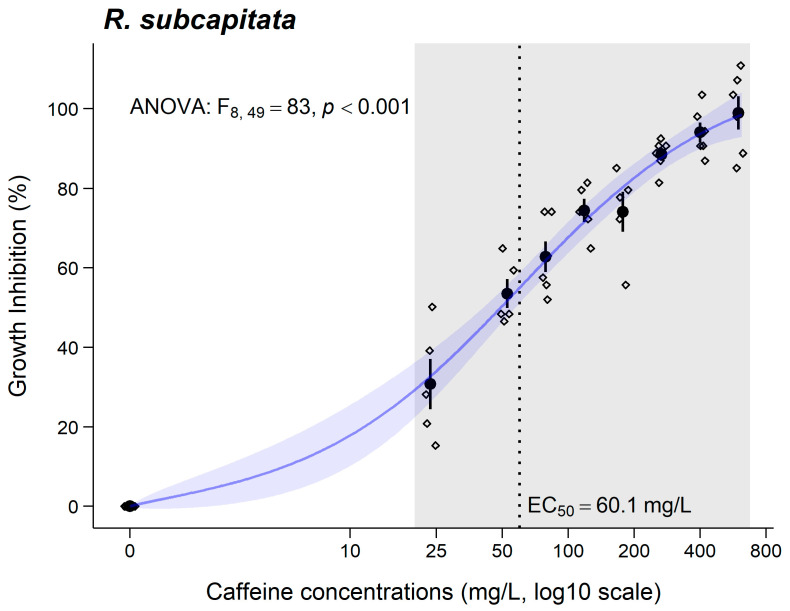
Results for growth inhibition (mean ± SE) in *R. subcapitata* after 72 h of exposure to caffeine. ° stands for individual values (replicates). Grey shadows stand for significant differences compared to the control group (0 mg/L) (Dunnett’s test, *p* < 0.05). EC_50_ value is also presented.

A significant increase in the growth inhibition of *R. subcapitata* was observed from the lowest concentration tested (23.4 mg/L; Figure 1). These results demonstrate that a concentration-dependent effect of CAF was also observed for *R. subcapitata*. The EC_50_ and respective CI_95%_ are represented in Figure 1 and Table 2.

### 3.3. Lemna minor

**Figure 2:**
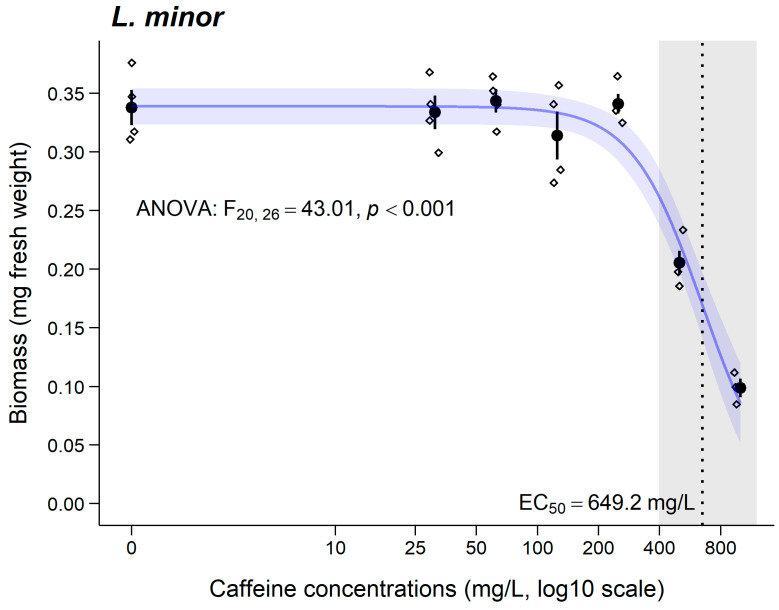
Results for biomass (fresh weight; mean ± SE) of *L. minor* after 7 days of exposure to caffeine. ° stands for individual values (replicates). Grey shadows stand for significant differences compared to the control group (0 mg/L) (Dunnett’s test, *p* < 0.05). EC_50_ value is also presented.

A significant decrease in the fresh biomass was observed only at the two highest concentrations of CAF tested (≥500 mg CAF/L; Figure 2). The EC_50_ and respective CI_95%_ are represented in Figure 2 and Table 2.

**Figure 3:**
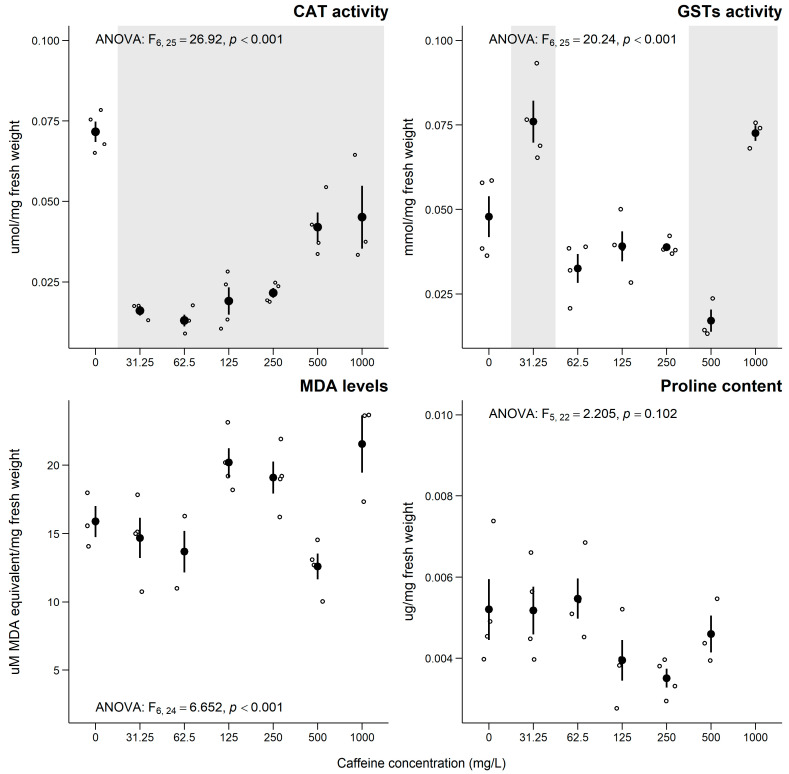
Results for sub-individual parameters (mean ± SE) in *L. minor* after 7 days of exposure to caffeine. ° stands for individual values (replicates). Grey shadows stand for significant differences compared to the control group (0 mg/L) (Dunnett’s test, *p* < 0.05). The lack of results in proline content at the highest concentration is due to the absence of *L. minor* biomass.

For the pigment contents, no significant effects were observed for the total chlorophyll (F_[6, 23]_ = 1.512; *p* = 0.233) and carotenoids (F_[5, 23]_ = 2.381; *p* = 0.080) contents. Regarding the biochemical biomarkers analyzed in *L. minor* after exposure to CAF, a significant decrease in CAT activity was observed at all tested concentrations (Figure 3). For the GSTs activity, the response was non-monotonic, showing a significant increase at concentrations of 31.3 and 1000 mg of CAF/L, while a significant decrease was noted at 500 mg of CAF/L (Figure 3). Exposure to CAF did not induce significant effects on the proline content or MDA levels at the tested concentrations of CAF (Figure 3).

### 3.4. Chironomus riparius

**Figure 4:**
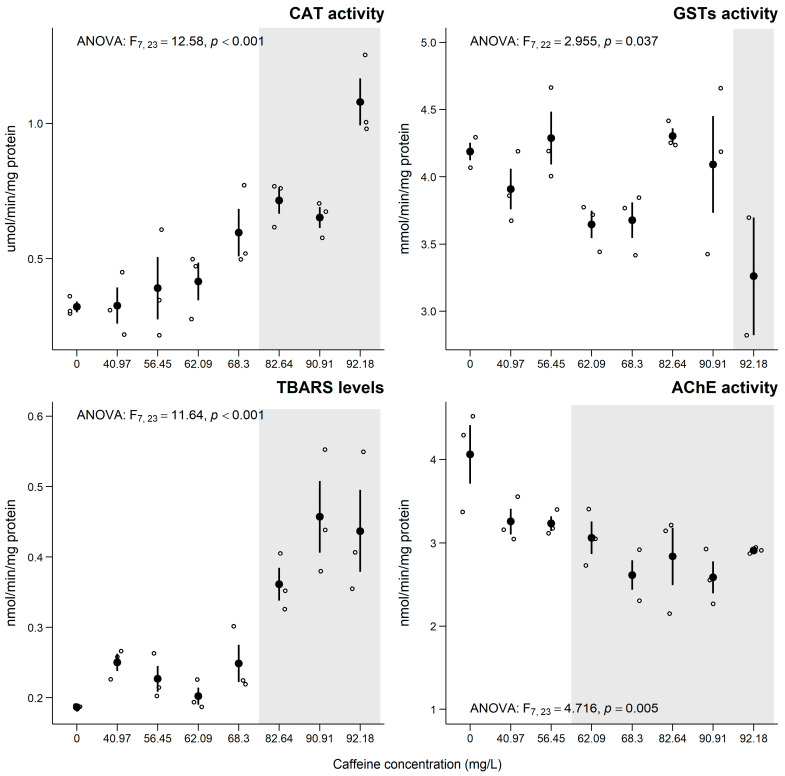
Results for biochemical biomarkers (mean ± SE) in *C. riparius* larvae after 10 days of exposure to caffeine. ° stands for individual values (replicates). Grey shadows stand for significant differences between concentration and the control group (0 mg/L) (Dunnett’s test, *p* < 0.05).

An increase in mortality of *C. riparius* larvae was observed across the tested concentrations for 1 day, with 100 % mortality at ≥ 1600 mg of CAF/L, with an LC_50_ = 644.5 mg of CAF/L (Table 2). The results for fresh biomass, following the simplified emergence assay, demonstrated that exposure to CAF induced a significant decrease, from 62.09 mg of CAF/L. For the sub-individual parameters analyzed in *C. riparius* after 10 days of exposure to CAF, a significant increase in CAT activity was observed at the three highest concentrations tested (≥ 82.64 mg/L; Figure 4). Concerning GSTs activity, a significant decrease was noted only at the highest concentration tested (92.8 mg/L; Figure 4). Exposure to CAF also induced significant effects on TBARS levels, showing an increase at the three highest concentrations tested (≥ 82.64 mg/L; Figure 4). Neurotoxic effects were also observed, indicated by a significant decrease in AChE activity above 62.09 mg CAF/L (Figure 4).

### 3.5. Species Sensitivity Distribution Outcomes

**Figure 5:**
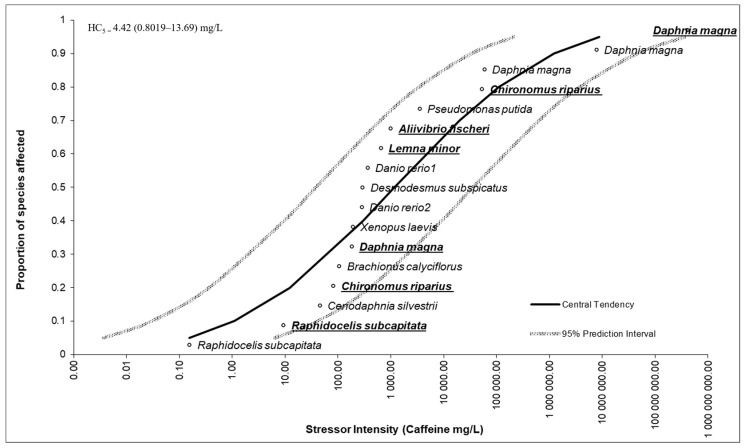
Species sensitivity distribution (SSD) plots, showing the distribution of EC_50_ values for organisms acutely exposed to CAF with 95% confidence intervals. Data were obtained from the literature, highlighted in bold and underlined for the present study (*Brachionus calyciflorus* [20]; *Ceriodaphnia silvestrii* [60]; *Danio rerio*^1^ [59]; *Danio rerio*^2^ [59]; *Daphnia magna* [28]; *Desmodesmus subspicatus* [61]; *Pseudomonas putida* [28]; *Raphidocelis subcapitata* [35]; *Xenopus laevis* [58]). Estimated hazardous concentrations for 5% of species (HC_5_) and respective lower (LL) and upper (UL) values are presented.

The SSD for freshwater species to CAF, considering the literature data and the here-obtained data for our research team, is shown in Figure 5 [20,28,35,58,59,60].

HC_5_ and respective LL and UL values determined for CAF are also presented in Figure 5. The results demonstrated that concentrations ≤4.42 (0.80–13.69) mg/L will protect 95% of the mentioned freshwater species.

## 4. Discussion

### 4.1. Aliivibrio fischeri

*A. fischeri* is a bioluminescent marine bacterium that emits light under environmentally favorable conditions (e.g., oxygen concentration above 0.5 mg/L and absence of contaminants) [34]. Therefore, the presence of toxic compounds and environmental stressors compromises the luminescence emission of the bacteria [34,62]. This fact induces disturbances in cellular respiration, as the bioluminescence pathway is linked to the electron transport system in cellular respiration [34]. According to the results obtained in this study, exposure to CAF significantly inhibited bioluminescence (Table 2). This inhibition of bioluminescence may be associated with the inhibition of the enzyme luciferase (an enzyme that reacts with ATP and oxygen molecules to produce bioluminescence in various organisms), altering the ATP levels and thereby influencing light expression [62,63]. This indicates that CAF interferes with the biochemical processes involved in light production. Aguirre-Martínez et al. [29] also observed a significant decrease in the bioluminescence of *A. fischeri* after exposure to concentrations between 200 and 18,000 mg/L of CAF, coinciding with some concentrations tested in the present study (76.8 to 2252.3 mg/L of CAF). Lomba et al. [34] suggested that the mechanism of action of CAF is complex, considering the various impacts that can be observed. Regarding the cellular mechanism, the mode of action may be related to the inhibition of the enzyme phosphodiesterase [34,64]. Callahan et al. [64] describe that this enzyme plays a significant role in the production of bioluminescence in *A. fischeri*. Thus, when this enzyme is inhibited, the luminescence decreases, which could explain the results obtained. Concerning the acute toxicity of CAF to *A. fischeri*, the results obtained corroborate those observed by Lomba et al. [34], who reported an EC_50_ value of 1244.3 mg/L (anhydrous CAF, 99.5%), which is close to the value obtained in the present study, and classified CAF as nontoxic to this species (Table 2).

### 4.2. Raphidocelis subcapitata

*R. subcapitata* is considered an excellent biological model due to its role in nutrient recycling, ease of handling and cultivation, and its presence in all freshwater aquatic environments, as well as its sensitivity to chemical compounds [65,66]. Therefore, any effect on the development and growth of these organisms can compromise the proper functioning of aquatic ecosystems, leading to ecological consequences [67]. In this study, a significant decrease in the growth of *R. subcapitata* was observed after exposure to CAF. These results allowed the classification of CAF as hazardous for *R. subcapitata* (EC_50_ = 60.1 mg/L; Figure 1) by EU Directive 93/677/ECC [56]. In the study by Lomba et al. [34], an EC_50_ of 870.3 ± 3.25 mg/L (72 h) was reported, which is higher than the value observed in the present study. They further emphasized that higher concentrations of CAF correspond to greater toxicity and potentially lower chlorophyll content. Diniz et al. [35] exposed *R. subcapitata* to CAF during all growth phases and observed a significant decrease in biomass at all tested concentrations (15 to 1000 µg/L), determining an EC_50_ of 154.9 µg/L (16 days of exposure). It is important to note that the exposure duration to CAF in the study by Diniz et al. [35] differs from that in the present study, and exposure time is a factor capable of altering the metabolic pathways and physiological functions of *R. subcapitata*, thus affecting growth. Crane et al. [68] observed similar results, where CAF induced more severe effects in chronic exposures than in acute exposures in *Pseudokirchneriella subcapitata*. Aguirre-Martínez et al. [29] reported an inhibition of cellular growth in the microalgae *Isochrysis galbana* and *P. subcapitata* when exposed to concentrations of CAF at 100 and 500 mg/L, respectively, over 72 to 96 h. Conversely, Zarrelli et al. [20] found that concentrations of 150 mg/L of CAF did not affect the growth rate of *P. subcapitata* during the exponential phase, after 72 h of exposure. Consequently, the decrease in biomass of microalgae after exposure to CAF may result from interference with various metabolic processes, including photosynthesis, respiration, and cellular redox balance [35,69,70,71]. For example, changes in cellular processes, such as photosynthesis, respiration, and DNA replication can lead to reduced growth rates [71] and induce the production of reactive oxygen species (ROS), promoting oxidative stress that can damage proteins, lipids, and nucleic acids, compromising cellular viability and growth [35]. CAF may also impair chloroplast function, decreasing energy production and limiting microalgal growth [69,70]. It can disrupt nutrient absorption [35] and interfere with the cell osmotic balance, which affects ion transport and cellular volume regulation. These disruptions may collectively lead to a decrease in cell division rates and overall growth [69,70]. Diniz et al. [35] further emphasized that an excessive production of ROS induced by CAF can be lethal to cellular organelles due to oxidative processes.

### 4.3. Lemna minor

Regarding the results of the *L. minor* assay, a significant decrease in biomass (fresh weight) was observed at CAF concentrations above 500 mg/L, and the results allowed for the calculation of an EC_50_ value of 649.2 mg/L (Figure 2), classifying CAF as dangerous according to EU Directive 93/677/ECC [56]. Ramírez-Morales et al. [36] did not observe significant effects on the growth of *L. minor* after exposure to up to 200 mg/L of CAF, indicating that the EC_50_ value must be higher than that concentration, which corroborates the results obtained in the present study (Figure 2). However, Ramírez-Morales et al. [36] mentioned that, although they did not observe significant effects in *L. minor*, long-term effects or effects at other concentrations could occur. In this study, the concentrations were higher than the environmental concentrations previously reported in the literature [72,73], aiming to understand how *L. minor* responds in the presence of CAF residues.

Regarding the photosynthetic performance of *L. minor*, the pigment content (chlorophylls and carotenoids) was not significantly affected after exposure to CAF, demonstrating that the photosynthetic performance was not compromised. Changes in chlorophyll content can indicate the physiological state of the organism, and evaluating its concentration is a common tool in ecotoxicological studies on various chemical compounds [74,75]. Fekete-Kertésk et al. [76] reported that CAF concentrations (0.01, 0.1, 1, 10, and 100 mg/L) significantly inhibited chlorophyll pigment content in *L. minor*, reflecting reduced growth and a decrease in biomass. This inhibition of pigment content and the reduction in growth may be associated with decreased chloroplast activity, resulting in lower energy production for various metabolic processes [69,70].

Regarding the sub-individual parameters of *L. minor*, significant effects were observed upon exposure to CAF. CAT is an enzyme responsible for removing hydrogen peroxide (H_2_O_2_) from cells, a product of photorespiration produced in peroxisomes [77]. Changes in CAT activity are used as a tool for evaluating enzymatic antioxidant defense [4,78,79]. In this study, a significant decrease in CAT activity was observed at all tested CAF concentrations (Figure 3). Although this may suggest a disturbance in redox homeostasis, the absence of significant changes in proline content and MDA levels (Figure 3) indicates that oxidative stress was likely not fully established. As previously reported, the decreases in CAT activity in plants may result from excessive ROS (including H_2_O_2_), where antioxidant defenses (such as CAT) can become overwhelmed, leading to inactivation or reduced efficiency [80]. The reduced CAT activity may reflect an early inactivation or inhibition of the antioxidant defense system, possibly due to protein oxidation or direct enzyme interference by CAF or its metabolites [2,80]. CAF may also compromise the expression of the genes involved in antioxidant defense, including those encoding CAT [80]. It has also been reported that CAF can alter protein structures through oxidation, causing variations in enzyme conformation and, thus, affecting its catalytic function [80]. Additionally, CAF and its by-products may act as inhibitors of CAT, binding to the active site or altering configuration in such a way that the enzyme becomes less effective in converting H_2_O_2_ into water and oxygen [80]. According to Diogo et al. [19], who assessed the effect of CAF on *Danio rerio*, the decrease in CAT activity observed was explained by the increase in ROS production and the cell’s difficulty in removing H_2_O_2_, as well as potential inactivation or reduced efficiency due to oxidation of protein structures. Regarding the activity of GSTs, these enzymes facilitate the conjugation of electrophilic compounds with glutathione (GSH), making them easier to excrete and playing a crucial role in detoxification pathways for toxic compounds such as polycyclic hydrocarbons, pesticides, and pharmaceuticals [81]. The expression and activity of GSTs are extremely relevant, given their detoxification function, protection against oxidative stress, signaling (influencing the levels of hormones important for growth, development, and stress response), cellular regulation, and tolerance to environmental stress [82,83]. In this study, a significant increase in GSTs activity was recorded at the first (31.25 mg/L) and last (1000 mg/L) concentrations of CAF tested, while a significant decrease was observed at the 500 mg/L concentration (Figure 3). Although there is no clear trend, the increase in GSTs activity may have occurred as an adaptation of *L. minor* to exposure to the compound, representing its role in antioxidant defense and detoxification. On the other hand, the decrease in GSTs activity may suggest an imbalance in redox regulation or possible enzyme inhibition. While GSTs are typically upregulated in response to oxidative stress, under certain conditions, excessive ROS levels or direct molecular interference may impair their function [78]. However, given that no significant changes were observed in the MDA levels or proline content, the establishment of oxidative stress cannot be confirmed. Previous studies [36,84,85] suggested that proline may be involved in the energy metabolism of plant cells, and an increase in proline could provide energy in more demanding situations. However, under the conditions tested in this study, no changes in proline content were observed, which may indicate that the antioxidant and osmoregulatory defense mechanisms of the plant were sufficiently effective, keeping the baseline levels unchanged [86]. Finally, the levels of MDA, a product of the degradation of polyunsaturated lipids that can occur during lipid peroxidation in plant cells [45], did not show significant changes after exposure to CAF. This indicates that there was no lipid peroxidation, which suggests that antioxidant and detoxification defenses were effective against exposure to the tested CAF concentrations.

### 4.4. Chironomus riparius

In the acute exposure (24 h) with *C. riparius* larvae, an increase in mortality was observed, with 100 % mortality occurring at a concentration of 1600 mg/L of CAF. These results allowed for the calculation of an LC_50_ of 644.5 mg/L after 24 h of exposure to CAF (Table 2). These results corroborate the work of Moore et al. [3], who observed 100 % mortality of *Chironomus dilutus* after 48 h of exposure to 2000 mg CAF/L. However, it should be noted that the exposure time and the concentrations of CAF used were different from the present study. Moore et al. [3] determined an LC_50_ of 1233 ± 159 mg/L for *C. dilutus* after 48 h of exposure. These differences may be associated with the different tolerances of the species (*C. riparius* vs. *C. dilutus*) or the differences in the larval stage used. In this study, the larvae were in stage I (2 to 3 days old), while the larvae used in the study by Moore et al. [3] were in stage III (13 to 15 days old). Regarding the emergence assay (10 days), a significant decrease in biomass was observed starting from exposure to 62.09 mg CAF/L. This decrease may be due to alterations in the metabolic processes of the larvae, leading to a dysfunction in energy use, particularly through mitochondrial dysfunction and altered adenosine triphosphate (ATP) production [3]. For example, exposure to CAF may alter the production of ATP, the main energy source for cells, which can compromise the growth and development of the larvae [3]. On the other hand, exposure to CAF appears to induce oxidative stress in the exposed organisms (e.g., *Lemna minor* and *C. riparius* in this study), influencing their growth and biomass [3]. The toxic effects of CAF are often attributed to the interference with specific molecular targets, such as phosphodiesterases (PDEs), which regulate cyclic nucleotide signaling (cAMP and cGMP). Caffeine is a known non-selective PDE inhibitor, leading to increased intracellular cAMP levels and altered cell signaling [64]. Additionally, caffeine can induce mitochondrial dysfunction, resulting in elevated ROS production and oxidative stress, as reflected in our study by the increase of CAT and TBARS levels and altered GSTs activity (Figure 4). These mechanisms may hinder nutrient assimilation and biosynthetic processes, ultimately affecting larval growth and biomass accumulation [30,31,32]. Moore et al. [3] also demonstrated that exposure to CAF induces damage to the digestive system of *C. dilutus*, compromising its ability to absorb nutrients and, consequently, affecting growth. Regarding biochemical determinations, significant effects were observed after the 10-day exposure of *C. riparius* to CAF. A significant increase in CAT activity was observed at the three highest concentrations tested (82.64, 90.91, and 92.18 mg CAF/L), while a decrease in GSTs activity was noted only at the highest concentration (92.18 mg CAF/L) (Figure 4). The increase in CAT activity may have resulted from an antioxidant response of the organism to combat the increase in ROS, specifically H_2_O_2_. The decrease in GSTs activity may have resulted from the organism’s reduced ability to detoxify and eliminate CAF. Cruz et al. [30] also observed a significant increase in CAT activity in the marine mollusk *Ruditapes philippinarum* exposed to 3.0 and 18.0 µg CAF/L. Pires et al. [31] noted the same trend in marine polychaetes *Hediste diversicolor* exposed to the same concentrations of CAF (3.0 and 18.0 µg/L). Various authors have observed that in the presence of CAF, some organisms tend to increase their metabolic activity (such as enzymes involved in antioxidant defense and detoxification) while reducing energy reserves used to combat oxidative stress [30,31,32]. TBARS are compounds formed as byproducts of lipid peroxidation and allow for the assessment of oxidative damage that the compounds may induce at the level of cellular membranes. In this study, the levels of TBARS at the higher concentrations (>82.64 mg CAF/L) indicated an increase in lipid peroxidation, suggesting that the organism is under oxidative stress and indicating cellular damage [87]. However, Diogo et al. [19] did not record oxidative damage in juveniles of *D. rerio* after 28 days of exposure to CAF (0.16–50 µg/L). These results indicate the differences in sensitivities between species when exposed to CAF, demonstrating the need for multi-species risk analysis studies.

Although the present study did not assess the absorption, biotransformation, or elimination of caffeine in the test organisms, several mechanisms can be inferred from previous studies. CAF is highly water-soluble (20 g/L) and has a low log K_ow_ (−0.07), facilitating passive diffusion across biological membranes in aquatic organisms [1,5]. In invertebrates such as *C. riparius*, absorption is likely to occur via the integument and gill-like structures, while in plants such as *L. minor*, uptake may occur through the roots and fronds via apoplastic and symplastic pathways. Although biotransformation pathways in invertebrates (and plants) remain poorly characterized, studies in fish and mollusks have demonstrated the phase I oxidative metabolism of CAF via cytochrome P450 enzymes, producing metabolites such as paraxanthine, theobromine, and theophylline, some of which may possess their toxicological profiles [1,19,27,28,29,30,31].

Regarding AChE activity, significant inhibition was observed starting from 62.09 mg CAF/L (Figure 4). Diogo et al. [19], in the exposure of *D. rerio* (28 days) to CAF, also observed a significant decrease in the AChE levels at the two highest concentrations tested (19.23 and 50 µg/L). AChE is an enzyme that acts in cholinergic neurotransmission, which is responsible for degrading the neurotransmitter acetylcholine (ACh) at synapses and neuromuscular junctions [87]. CAF can prevent the binding of adenosine to the receptor [88], indirectly causing the release of neurotransmitters such as dopamine, gamma-aminobutyric acid (GABA), and glutamate, disrupting cellular neurotransmission [89]. Thus, CAF modulates neurotransmitter release and cellular excitability [15,19], which can explain the observed inhibition of AChE activity in *C. riparius*, indicating disturbances in the cholinergic neurotransmission. This potential accumulation of ACh in the synapse may induce prolonged hyperstimulation of nerve and muscle fibers, affecting locomotor activities and compromising normal behavior, including feeding, escape response, and reproduction [15].

### 4.5. Species Sensitivity Distribution Outcomes

Figure 5 presents the species sensitivity distribution (SSD) constructed with the literature EC_50_ data for different aquatic organisms acutely exposed to CAF, including those obtained in the present study. The central tendency curve and 95% prediction intervals indicate a wide variation in sensitivity among species, with the EC_50_ values ranging from concentrations below milligrams (µg/L) to values greater than 10,000 mg/L. The results reveal that *R. subcapitata* was the most sensitive species to CAF, with an EC_50_ below 1 mg/L, while species such as *D. magna*, in different studies, presented a wide variation in sensitivity, with much higher EC_50_ values, indicating low acute toxicity of this compound for these organisms.

Among the organisms included in this study, it was observed that the organisms presented varied sensitivities, being located in different positions along the curve. This distribution reinforces the importance of considering organisms from different trophic levels and taxonomic groups for the ecotoxicological evaluation of emerging contaminants, such as CAF, in natural ecosystems. The position of the data from this study within the SSD prediction range suggests consistency with the previously published data, validating the experimental approach used. The estimate of HC_5_ could be extracted from this distribution to support the ecological risk assessment processes. The HC_5_ value estimated for CAF was 4.42 mg/L, with confidence limits ranging from 0.80 to 13.69 mg/L. This concentration represents the threshold below which 95% of aquatic species are expected to be protected, according to the SSD approach. When compared to the environmental concentrations reported in the literature, ranging from ng/L to tens of µg/L across various aquatic compartments, the HC_5_ appears to be several orders of magnitude higher. However, maximum environmental concentrations reported in effluents can reach up to 86 mg/L [17,18], and values above 30 µg/L have been detected in surface waters [11,12,15] and drinking water [13,14]. Although these concentrations are still below the HC_5_, they fall within or above the lower confidence bound (0.80 mg/L), raising concerns for sensitive species under chronic exposure scenarios or in ecosystems subject to continuous inputs. Moreover, sublethal effects at the biochemical and behavioral levels, not captured by the HC_5_ estimates based on individual endpoints, may occur at much lower concentrations, potentially compromising ecosystem health in the long term.

### 4.6. Implications for Environmental Risk Assessment and Regulatory Context

The findings of this study contribute to the growing evidence that caffeine, although generally classified as a low-priority contaminant due to its low bioaccumulation potential and moderate acute toxicity, can elicit sub-lethal effects in aquatic organisms, including metabolic disturbances, oxidative stress, and neurotoxicity. According to the EU Directive 93/67/EEC, CAF was classified in our study as hazardous for *R. subcapitata* and *C. riparius*, and nontoxic for *A. fischeri* and *L. minor* based on the E(L)C_50_ values. However, this classification does not account for sub-individual effects or chronic exposure, which may occur at significantly lower concentrations. The E(L)C_50_ values obtained in this study provide useful information for hazard classification and inter-species comparison but should be interpreted with caution in regulatory contexts. First, they reflect acute or short-term exposure and may underestimate long-term ecological risks. Second, species-specific sensitivity may vary according to the developmental stage, environmental conditions, and mixture effects. Finally, the E(L)C_50_ values based on growth or mortality do not capture more subtle but ecologically relevant effects, such as those observed in our sub-individual biomarker analyses.

CAF is widely recognized as a contaminant of emerging concern due to its high detection frequency in aquatic systems, even in wastewater treatment effluents [1,10,11]. Although it is not currently included in monitoring programs under the EU Water Framework Directive, it is often used as a marker for anthropogenic pollution and wastewater discharge [2,16]. It is important to contextualize the concentrations tested in this study with respect to the environmental occurrences of CAF. According to the literature reports, CAF concentrations in surface waters typically range from 0.05 to 33.2 µg/L [11,12], with values in drinking water reaching up to 35 µg/L [13,14] and in wastewater effluents as high as 86 mg/L [17,18]. Although the nominal concentrations used in this study exceed most-reported environmental levels, they are consistent with the worst-case scenarios associated with untreated effluents, high-consumption periods, or inadequate removal in wastewater treatment plants. Furthermore, these concentrations are necessary to determine the E(L)C_50_ values that support hazard classification and SSD modeling. Notably, sub-individual alterations, such as oxidative stress and neurotoxicity in *L. minor* and *C. riparius,* were observed at concentrations starting from 31.25 mg/L to 62.09 mg/L, respectively. These values, while above-average surface water levels, are still below the maximum concentrations recorded in some effluent samples and overlap the lower confidence bound (0.80 mg/L) of the estimated HC_5_ (4.42 mg/L). This reinforces the concern for sensitive species in ecosystems subject to chronic exposure or continuous CAF input. These findings support the recommendation for the environmental monitoring of CAF, particularly in densely populated or poorly treated areas.

Although the current study focused on acute and sub-chronic exposures, for future research, we recommend long-term experiments at environmentally relevant concentrations, the inclusion of mixture exposures with other commonly detected contaminants (e.g., pharmaceuticals or pesticides), and a focus on population- and community-level effects. Moreover, mechanistic studies that explore transcriptomic or metabolomic changes can help clarify the mode of action of CAF in non-target species. Such studies will enhance the robustness of ecological risk assessments and inform potential regulatory updates regarding emerging contaminants like caffeine.

## 5. Conclusions

Among the tested species, *R. subcapitata* was the most sensitive to CAF exposure, with growth inhibition observed from 23.4 mg/L. This was followed by *C. riparius* (effects on survival, biomass, and biochemical markers from 62 mg/L) and *L. minor* (biomass, at ≥ 500 mg/L, and oxidative stress biomarkers at ≥ 31.25 mg/L), and the least-sensitive species in this work was *A. fischeri* (bioluminescence inhibition). These results also demonstrate that CAF can cause damage to metabolic pathways, alter physiological functions such as neurotransmission, and compromise the growth and mobility of organisms, potentially leading to mortality. Given the observed sub-individual effects and their potential ecological consequences, we strongly recommend that future studies address long-term, low-level exposures to CAF under realistic environmental conditions. This will allow for better assessment of chronic toxicity, mixture effects, and the resilience of aquatic communities to continuous contaminant inputs. On the other hand, caffeine is already considered an emerging contaminant of concern, so it is crucial to understand how its potential toxicity to non-target organisms may be altered in relevant environmental contexts and scenarios of climate change.

## Figures and Tables

**Table 1:** Experimental conditions for CAF exposure to *A. fischeri*, *R. subcapitata*, *L. minor*, and *C. riparius*.

	*A. fischeri*	*R. subcapitata*	*L. minor*	*C. riparius*	
Exposure time	30 min	3 days	7 days	1 day	10 days
Endpoint	Bioluminescence inhibition	Growth inhibition	Biomass (fresh weight)	Mortality	Biomass (fresh weight)
Culture medium	Distilled water	Woods Hole MBL	Steinberg	ASTM hard water	
Stock solution (mg/L)	2750	600	1000	2000	3000
Nominal [CAF] (mg/L)	4.8–2252 (dilution factor 2×)	23.4–600 (dilution factor 1.5×)	31.25–1000 (dilution factor 2×)	268.4–2000 (dilution factor 1.25×)	40.97–92.18 (dilution factor 1.1× and 1.5×)
Real-lowest and highest [CAF] (mg/L)	6.0 and 2191.0	22.0 and 692.0	31.0 and 1043.0	206.0 and 1828.0	36.0 and 88.0

**Table 2:** Results of E(L)C50 and respective 95 % confidence intervals (CI95 %) after CAF exposure to *A. fischeri*, *R. subcapitata*, *L. minor*, and *C. riparius*, and respective toxicity classes according to the EU-Directive 93/677/ECC (EC 1996): very toxic (EC_50_ < 1 mg/L), toxic (EC_50_: 1–10 mg/L), hazardous (EC_50_: 10–100 mg/L), and nontoxic (EC_50_ > 100 mg/L). The limit of quantification (LOQ) was 0.00001 mg/L for CAF.

	*A. fischeri*	*R. subcapitata*	*L. minor*	*C. riparius*	
E(L)C_50_ (CI_95%_) (mg/L)	30 min 998.5 (329.9–1667)	3 days 60.1 (34.89–85.33)	7 days 649.2 (557.8–740.6)	1 day LC_50_ = 644.5 (578.4–710.6)	10 days 81.62 (74.27–88.97)
Toxicity class	Nontoxic	Hazardous	Nontoxic	-	Hazardous

## Data Availability

The original contributions presented in this study are included in the article. Further inquiries can be directed to the corresponding author(s).
